# Effects of Expanding Direct-to-Consumer Alcohol Home Delivery Policies: Evidence From 18 States of Increases in Alcohol Use and Consequences

**DOI:** 10.15288/jsad.24-00273

**Published:** 2025-05-06

**Authors:** Jimikaye B. Courtney, McKenna Roudebush, Rebecca S. Williams, Melissa J. Cox, Kurt M. Ribisl

**Affiliations:** ^a^ Department of Exercise and Sport Science, University of North Carolina at Chapel Hill, Chapel Hill, North Carolina; ^b^ UNC Lineberger Comprehensive Cancer Center, University of North Carolina at Chapel Hill, Chapel Hill, North Carolina; ^c^ Center for Health Promotion and Disease Prevention, University of North Carolina at Chapel Hill, Chapel Hill, North Carolina; ^d^ Department of Health Behavior, Gillings School of Global Public Health, University of North Carolina at Chapel Hill, Chapel Hill, North Carolina

## Abstract

**Objective:**

After the COVID-19 pandemic onset, several U.S. states passed legislation to begin or expand direct-to-consumer (DTC) alcohol home delivery. We examined changes in DTC use and associations between DTC use, drinking patterns, and negative consequences by different DTC policies.

**Method:**

We conducted a cross-sectional, retrospective survey of 5,360 U.S. adults who consumed alcohol (ages 21–62 years), using an online Qualtrics survey panel. Participants were recruited from 18 states representing four DTC policy groupings pertaining to on- and off-premises outlets (no DTC, no change in existing DTC policy, new DTC policy, expanded DTC policy). DTC use and drinking patterns (average drinks/week, binge drinking days/month) were self-reported for four pandemic-related periods at pre- (2019–February 2020), early (March–May 2020), mid- (June 2020–March 2021), and late pandemic (April 2021–October 2023). Participants self-reported the total number of negative drinking consequences (out of 17) they experienced during the entire pandemic. Multivariate regressions examined time and policy group predicting DTC use, time and DTC use predicting drinking patterns, and DTC use and drinks/week predicting negative consequences.

**Results:**

Compared to adults in states with no delivery, odds of DTC use were highest among adults in states that expanded DTC policies (odds ratio [OR] = 2.11, 95% confidence interval [CI] [1.78, 2.50]). DTC use was associated with consuming approximately 4.43 (*p* < .001) more average drinks per week, more binge days per month (incidence rate ratio [IRR] = 1.33, 95% CI [1.27, 1.39]), and more negative consequences (IRR = 1.28, 95% CI [1.17, 1.39]), controlling for current drinking.

**Conclusions:**

Our findings suggest that expanding DTC delivery policies increased alcohol accessibility, and DTC use was associated with increases in excessive alcohol use and consequences. Such data can inform future decisions about states' DTC alcohol policies.

One in six u.s. adults report binge drinking in the past 30 days, 25% of whom consume at least eight drinks per binge drinking occasion (Centers for Disease Control and Prevention [CDC], 2022). Excessive alcohol use accounted for more than 178,000 deaths annually during 2020–2021 (Esser et al., 2024). In addition, excessive alcohol use cost the United States $249 billion in 2010 (most recent estimate available), with 40% of costs carried by state and federal governments (Sacks et al., 2015). These costs were attributable to lost productivity (71.9%), health care (11.4%), and other (16.7%) costs (Sacks et al., 2015). To effectively reduce these health and economic costs, states can decrease excessive alcohol use through implementation of evidence-based alcohol control policies (Babor et al., 2022).

Alcohol policies affect alcohol use by impacting availability of and access to alcohol (Babor et al., 2022; Community Preventive Services Task Force, 2023). The World Health Organization (WHO) identified policies to restrict alcohol availability as an essential strategy for reducing alcohol-related consequences, citing the ability to purchase alcohol for direct-to-consumer (DTC) delivery as an emerging public health challenge (WHO, 2019). In this study, DTC delivery refers to when alcohol is delivered directly to a person's home from an online vendor or physical establishment (e.g., bar or restaurant). DTC delivery sales raised early concerns about youth access to alcohol. A 2011 study documented inadequate age verification procedures by online alcohol vendors, which permitted 45% of DTC delivery orders placed by underage buyers to be received (Williams & Ribisl, 2012). Concerns about potential negative consequences associated with DTC delivery have broadened to include concerns about alcohol-related consequences among adults who use DTC (Gray Phillip et al., 2018; Mojica-Perez et al., 2019).

The availability of DTC alcohol delivery has expanded worldwide, increasing alcohol accessibility (Gray Phillip et al., 2018) and potentially facilitating excessive alcohol use. For example, one Australian study found that 69% of people who ordered DTC alcohol via on-demand home delivery (order received in <2 hours) engaged in binge drinking, 28% consumed ≥11 standard drinks on the occasion for which they placed the order, and this group drank more heavily than people with slower order delivery (Mojica-Perez et al., 2019). More than 28% of the Australians in the study reported that delivery enabled the continuation of an ongoing drinking episode (Mojica-Perez et al., 2019). U.S. online alcohol sales accounted for about 4% of retail sales in 2020–2022, and this proportion has been increasing (Droesch, 2023).

The availability of DTC alcohol delivery has increased since the onset of the COVID-19 pandemic (hereafter, “the pandemic”) because of state policy changes in the United States (National Institute on Alcohol Abuse and Alcoholism [NIAAA], 2022; Trangenstein et al., 2023b). States initially enacted more permissive DTC delivery policies to provide economic support to the hospitality industry and to increase ease of access while people were engaging in COVID-19 mitigation practices (Colbert et al., 2021). Many states expanded DTC policies to permit off-premises outlets to deliver spirits and/or to permit on-premises establishments to deliver beer, wine, and spirits (NIAAA, 2022). Compared with before the pandemic onset in March 2020, during the pandemic, the number of people living in states that permitted DTC delivery increased by 284% (170.3 million people) (Trangenstein et al., 2023b).

The number of people purchasing alcohol online was three times higher in the middle 2 weeks of April 2020 (early pandemic) compared with the pre-pandemic period in the last 2 weeks of February 2020 (Nielsen, 2020). About 43% of people in the United States who drink ordered alcohol using DTC delivery for the first time during the pandemic (The International Wines and Spirits Record, 2021). Up to 21% of U.S. adults used DTC delivery at some point during the first year of the pandemic (Grossman et al., 2022; Trangenstein et al., 2023b), and they purchased larger volumes of alcohol compared with the year before the pandemic (Nielsen, 2020). One study found that U.S. adults who used DTC delivery during May 2020 consumed alcohol more frequently, in larger quantities, and had two times higher odds of binge drinking than those who accessed alcohol via other means (Grossman et al., 2022). In general, adults who used DTC delivery during the pandemic reported greater alcohol consumption and higher odds of excessive alcohol use than those who did not access alcohol via DTC delivery (Coomber et al., 2024; Huckle et al., 2021; MacNabb et al., 2022; Trangenstein et al., 2023a).

Existing studies examining DTC policies largely focus on the first year of the pandemic; however, the effects of these policies may be long-lasting. Indeed, many policy changes that were intended to be temporary have become permanent (Colbert et al., 2021; Lemp et al., 2024; NIAAA, 2022). It remains unclear how these changes have affected alcohol use, including after the COVID-19 public health emergency ended. Therefore, the purpose of this study was to examine the effects of time and DTC alcohol home delivery policies on the use of alcohol delivery and on alcohol use patterns and consequences across the extended time frame of prepandemic (2019) through late pandemic (October 2023).

## Method

### Participants and procedures

Cross-sectional, retrospective survey data were collected from a convenience sample of participants identified and recruited through the Qualtrics Online Survey Panel platform. Qualtrics emailed eligible individuals who were part of their existing panels for social science research to participate in the study. Data were collected from July 12 to October 31, 2023. Participants were eligible if they were age 21–62 years, were living in one of the 18 states selected for the study due to DTC policies and demographics (details below), and reported consuming 12 or more drinks/year during 2019 or during March 2020 to the time of the survey. Quotas were used to ensure that the sample was at least 40% male and had approximately equal representation for each state (278–304 participants/state). Eligible participants completed a single online survey. On average, the survey took 23 minutes to complete, and Qualtrics compensated each participant $8.

The survey was developed using a series of existing measures and researcher-generated items. The survey was edited by an alcohol policy advisory board, tested by study personnel, and fielded with a pilot sample of 100 participants, after which it was disseminated to all potential participants. The Checklist for Reporting Results in Internet E-Surveys (Eysenbach, 2004) is available in Supplemental Table A. (Supplemental material appears as an online-only addendum to this article on the journal's website.) All research procedures were approved as an exempt study by the University of North Carolina at Chapel Hill Institutional Review Board (#22-2093).

### Alcohol policy groups

An alcohol policy advisory board—comprised of 16 experts from government, private, and academic entities—virtually convened on three occasions to review survey questions and state DTC alcohol delivery policies across the United States. The research team identified commonalities and differences in DTC delivery policies across states before and after the pandemic onset to find groups of states with similar policies, which were reviewed and modified with the advisory board. State-level alcohol policies were identified using data from the Alcohol Policy Information System, which included updates on DTC alcohol delivery–related policies pertaining to sales from on-premises and off-premises establishments, with no specific information about online sales (NIAAA, 2022). There is wide variability in DTC alcohol delivery policies related to the type of alcohol, who may deliver alcohol, the type of alcohol container, and whether food must be ordered with alcohol for delivery from restaurants (NIAAA, 2022). However, for the purpose of this study, the research team and advisory board focused on whether DTC alcohol delivery was generally allowed or not. This process led to the creation of the following four DTC policy groups: (a) the No Delivery Allowed Group included states that did not permit any on- or off-premises alcohol delivery before or during the pandemic; (b) the No Change in Delivery Group included states that allowed on- and/or off-premises alcohol delivery before the pandemic and did not introduce any policy changes after the pandemic onset; (c) the New Delivery Group included states that did not permit any on- or off-premises alcohol delivery before the pandemic but made changes to allow on- and/or off-premises alcohol delivery after the pandemic onset; and (d) the Expanded Delivery Group included states that allowed on- and/or off-premises alcohol delivery before the pandemic and expanded delivery options and/or loosened restrictions after the pandemic onset (Supplemental Figure A). The team and board collaboratively selected 18 states to include in survey data collection. The aim was to have a representative sample of U.S. residents based on sociodemographic, geographical, and other considerations. The selected states included the following: (a) No Delivery Allowed Group: Delaware and Kansas (*n* = 587 participants); (b) No Change in Delivery Group: Idaho, Minnesota, and Rhode Island (*n* = 870); (c) New Delivery Group: Georgia, Montana, and New Mexico (*n* = 903); and (d) Expanded Delivery Group: Arkansas, California, Colorado, Florida, Illinois, Maine, Mississippi, New York, North Carolina, and Oregon (*n* = 3,000).

### Measures

*Survey time periods*. Participants retrospectively self-reported alcohol use patterns and use of DTC alcohol delivery for each of the following periods: (a) pre-pandemic (2019–February 2020), (b) early pandemic (March–May 2020), (c) mid-pandemic (June 2020–March 2021), and (d) late pandemic (April 2021–survey date [July 12–October 31, 2023]). These periods were defined at the beginning of the survey with an accompanying image that described key features of the periods. For example, early pandemic was described as “during lockdown periods,” and late pandemic was described as “post vaccinations becoming widely available.” Participants were reminded of these definitions three additional times to improve recall.

*Alcohol use measures*. The first two items from the Alcohol Use Disorders Identification Test were used to assess frequency and quantity of alcohol consumption (Bradley et al., 2003; National Institute on Drug Abuse, 2023). Participants were asked how frequently they typically consumed alcohol during each period (“drinking frequency”; *never, less than monthly, monthly, weekly, 2–3 times/week, 4–6 times/week, daily*) and how many drinks they consumed on a typical day (“drinking quantity”; *1, 2, 3, or 4 drinks, 5–6 drinks, 7–9 drinks, 10 or more drinks*), which were based on U.S. standard alcoholic drink sizes and accompanied by a picture of standard drinks (U.S. Department of Agriculture & U.S. Department of Health and Human Services, 2019). Drinking frequency was standardized to number of days per week. Average drinks per week were calculated as drinking days/week (drinking frequency) × drinks/drinking day (drinking quantity).

Frequency of binge drinking was assessed by asking participants to report the number of days per month (0–30 days) they engaged in binge drinking, defined as consuming ≥4 drinks for females or ≥5 drinks for males, on an occasion.

*Negative drinking consequences*. Negative drinking consequences were assessed for the “entire COVID-19 pandemic period” using the Short Inventory of Problems–Revised (SIP-R; Kiluk et al., 2013). The SIP-R includes 17 items assessing physical, social, intrapersonal, interpersonal, and impulse control problems associated with drinking alcohol (Kiluk et al., 2013). Participants responded “yes” or “no” to having experienced each of the 17 items during the entire pandemic, with “yes” responses summed to calculate the total number of negative consequences.

*DTC measures*. Participants answered, “How did you typically obtain the alcohol you drank,” selecting all applicable sources at each period using categories from the 2021 Summer Styles survey, administered by Porter Novelli, such as *liquor store, convenience store, supermarket, or gas station; restaurant, brewery, bar, or club;* and *had it delivered to me* (Porter Novelli, 2023). Participants who did not select “had it delivered to me” were categorized as not using DTC delivery during that period. Participants who selected “had it delivered to me” were categorized as using DTC delivery during that period and were subsequently asked how they accessed alcohol delivery using a single question that included six categories alongside a write-in response option. Example categories included “common delivery carrier like UPS/FedEx,” “delivery app/service focused on alcohol [e.g., Drizly, Saucey, Minibar],” and “a delivery app/service focused on restaurants or bars [e.g., Grubhub, UberEats, DoorDash].”

*Covariates*. Sociodemographic characteristics were collected using items from the PhenX Toolkit (Hamilton et al., 2011), including age in years, gender identity, ethnicity, race, education, annual income, marital status, and parental status. State-level alcohol policy scale scores were included as a measure of the overall restrictiveness of the alcohol policy environment (Blanchette et al., 2020).

### Statistical analysis

*Quality assurance*. Before analyses, responses underwent quality checks by Qualtrics and the research team. Qualtrics screened 13,720 potential participants. Of those, 6,535 (47.6%) were ineligible and 53 (0.4%) did not provide consent. Of the 7,132 eligible respondents who consented to participate, 6,006 (84.2%) completed the survey. Quality checks (e.g., duplicates based on internet protocol [IP] addresses and cookies, bots identified via ReCAPTCHA, and responses completed in less than 50% of the median completion time) resulted in the removal of 1,006 participants (16.7%), resulting in a final analytic sample of 5,360 survey respondents.

*Descriptive statistics*. Sociodemographic characteristics and descriptive statistics are presented for the entire sample. Continuous variables on alcohol use are reported as mean ± *SD*, median, and the 75th percentile. Categorical variables are reported as counts and percentages.

*Models*. Logistic regressions examined the effects of time and alcohol policy group on the odds of using DTC delivery. People in the No Delivery Allowed Group should presumably not have access to DTC delivery; however, 14.5% of people in that group reported using DTC delivery during at least one period. Therefore, models were run with and without the No Delivery Allowed Group included. The modeling approach used for examining the effects of time and use of DTC alcohol delivery on average drinks/week, binge drinking days/month, and the total number of negative drinking consequences varied by outcome variable. A linear regression model was used when average drinks/week was the outcome. A series of intercept-only models with different distributional assumptions (e.g., Poisson, zero-inflated Poisson, zero-inflated negative binomial) were estimated to identify the best models for the count-based binge drinking and negative consequences data and the functional form of time (i.e., linear, quadratic, cubic) in the models by comparing multiple fit indices (e.g., Akaike information criterion, log-likelihood ratio test; Everitt & Hothorn, 2010; Hilbe, 2011; Tüzen & Erba , 2018) (Supplemental Table B). Models included the effects of time, alcohol policy group, sociodemographic control variables (age, gender identity, race, ethnicity, education, income, alcohol policy scale score), and Time × Policy Group or Time × DTC Use interactions and, for negative consequences, Average Drinks/Week × DTC Use interactions. All nonsignificant interactions were removed for parsimony. Average drinks/week consumed across all four periods (average of pre-, early, mid-, and late pandemic drinks/week) was an additional covariate in the model with negative consequences as the outcome. For logistic, negative binomial, or zero-inflated negative binomial models, results are reported as odds ratios (ORs) and/or incidence rate ratios (IRRs) with 95% confidence intervals (CIs), and for Gaussian models, results are reported as unstandardized betas (*b*s), standard errors (*SE*s), and standardized betas (βs). All models were fitted in R Version 4.2.2 (Lumley, 2004; R Core Team, 2020).

## Results

### Sample characteristics

Adults in this sample (*N* = 5,360) were 46.9% male, 74.7% White, 13.9% Black, and 12.3% Hispanic (Table 1). Half of the adults were married (50.4%), and nearly half had one or more children living in their home during at least one of the four periods (48.4%–49.3%).

**Table 1.**
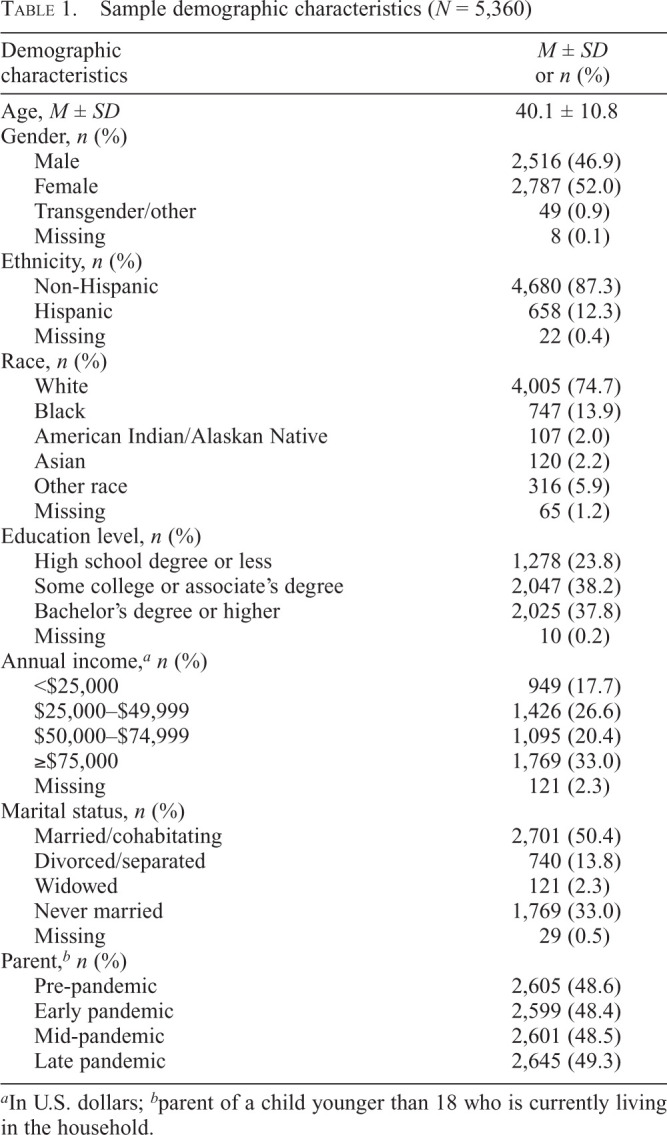
Sample demographic characteristics (*N* = 5,360)

Demographic characteristics	*M ± SD* or *n* (%)
Age, *M ± SD*	40.1 ± 10.8
Gender, *n* (%)	
Male	2,516 (46.9)
Female	2,787 (52.0)
Transgender/other	49 (0.9)
Missing	8 (0.1)
Ethnicity, *n* (%)	
Non-Hispanic	4,680 (87.3)
Hispanic	658 (12.3)
Missing	22 (0.4)
Race, *n* (%)	
White	4,005 (74.7)
Black	747 (13.9)
American Indian/Alaskan Native	107 (2.0)
Asian	120 (2.2) Other race 316 (5.9)
Missing	65 (1.2)
Education level, *n* (%)	
High school degree or less	1,278 (23.8)
Some college or associate's degree	2,047 (38.2)
Bachelor's degree or higher	2,025 (37.8)
Missing	10 (0.2)
Annual income,*^a^ n* (%)	
<$25,000	949 (17.7)
$25,000–$49,999	1,426 (26.6)
$50,000–$74,999	1,095 (20.4)
≥$75,000	1,769 (33.0)
Missing	121 (2.3)
Marital status, *n* (%)	
Married/cohabitating	2,701 (50.4)
Divorced/separated	740 (13.8)
Widowed	121 (2.3)
Never married	1,769 (33.0)
Missing	29 (0.5)
Parent,*^b^ n* (%)	
Pre-pandemic	2,605 (48.6)
Early pandemic	2,599 (48.4)
Mid-pandemic	2,601 (48.5)
Late pandemic	2,645 (49.3)

^a^ In U.S. dollars;

^b^ parent of a child younger than 18 who is currently living in the household.

Alcohol use in this sample increased during the early- and mid-pandemic periods and reduced slightly during the late pandemic, but not to pre-pandemic levels (Table 2). Respondents also reported the lowest number of binge drinking days/month during the pre-pandemic period and the highest during the mid-pandemic period. Overall, 25.2% of the sample used DTC alcohol delivery during at least one period, with participants predominantly using a delivery app/service focused on alcohol, such as Drizly (40.2%, merged under UberEats as of March 2024, after survey completion for this study) and/or food, such as GrubHub (43.1%).

**Table 2.**
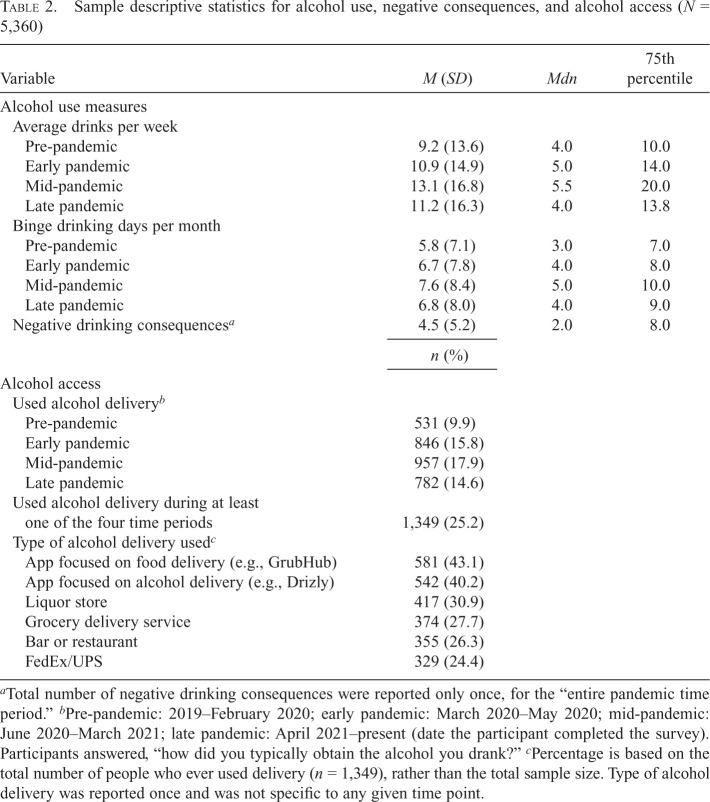
Sample descriptive statistics for alcohol use, negative consequences, and alcohol access (*N* = 5,360)

Variable	*M* (*SD*)	*Mdn*	75th percentile
Alcohol use measures			
Average drinks per week			
Pre-pandemic	9.2 (13.6)	4.0	10.0
Early pandemic	10.9 (14.9)	5.0	14.0
Mid-pandemic	13.1 (16.8)	5.5	20.0
Late pandemic	11.2 (16.3)	4.0	13.8
Binge drinking days per month			
Pre-pandemic	5.8 (7.1)	3.0	7.0
Early pandemic	6.7 (7.8)	4.0	8.0
Mid-pandemic	7.6 (8.4)	5.0	10.0
Late pandemic	6.8 (8.0)	4.0	9.0
Negative drinking consequences*^a^*	4.5 (5.2)	2.0	8.0

	*n* (%)		
Alcohol access			
Used alcohol delivery*^b^*			
Pre-pandemic	531 (9.9)		
Early pandemic	846 (15.8)		
Mid-pandemic	957 (17.9)		
Late pandemic	782 (14.6)		
Used alcohol delivery during at least one of the four time periods	1,349 (25.2)		
Type of alcohol delivery used*^c^*			
App focused on food delivery (e.g., GrubHub)	581 (43.1)		
App focused on alcohol delivery (e.g., Drizly)	542 (40.2)		
Liquor store	417 (30.9)		
Grocery delivery service	374 (27.7)		
Bar or restaurant	355 (26.3)		
FedEx/UPS	329 (24.4)		

^a^ Total number of negative drinking consequences were reported only once, for the “entire pandemic time period.”

^b^ Pre-pandemic: 2019–February 2020; early pandemic: March 2020–May 2020; mid-pandemic: June 2020–March 2021; late pandemic: April 2021–present (date the participant completed the survey). Participants answered, “how did you typically obtain the alcohol you drank?”

^c^ Percentage is based on the total number of people who ever used delivery (*n* = 1,349), rather than the total sample size. Type of alcohol delivery was reported once and was not specific to any given time point.

### Odds of using DTC alcohol delivery

Table 3 shows ORs from the logistic regressions with using versus not using DTC regressed on time and policy group. There was a quadratic (OR = 0.80, *p* < .001) effect of time such that the odds of using DTC gradually increased from pre-pandemic to mid-pandemic and then decreased into late pandemic (Supplemental Figure B). DTC policy group significantly predicted odds of using DTC alcohol delivery. Compared with the No Delivery Allowed Group, all other groups had higher odds of DTC use, including the No Change in Delivery Group (OR = 1.72, 95% CI [1.42, 2.09]), the New Delivery Group (OR = 1.55, 95% CI [1.28, 1.88]), and the Expanded Delivery Group (OR = 2.11, 95% CI [1.78, 2.50]). The Expanded Delivery Group had higher odds of DTC use than the New Delivery Group (OR = 1.22, 95% CI [1.09, 1.36]) and the No Change in Delivery Group (OR = 1.36, 95% CI [1.21, 1.52]). There were no significant Time × Policy Group interactions. As shown in Supplemental Table C, the results did not change with the No Delivery Allowed Group removed from the model.

**Table 3.**
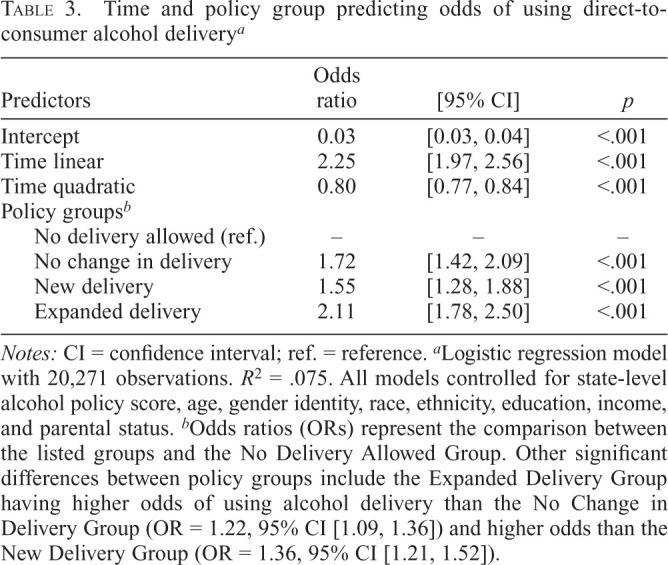
Time and policy group predicting odds of using direct-to-consumer alcohol delivery*^a^*

Predictors	Odds ratio	[95% CI]	*p*
Intercept	0.03	[0.03, 0.04]	<.001
Time linear	2.25	[1.97, 2.56]	<.001
Time quadratic	0.80	[0.77, 0.84]	<.001
Policy groups*^b^*			
No delivery allowed (ref.)	–	–	–
No change in delivery	1.72	[1.42, 2.09]	<.001
New delivery	1.55	[1.28, 1.88]	<.001
Expanded delivery	2.11	[1.78, 2.50]	<.001

*Notes:* CI = confidence interval; ref. = reference.

^a^ Logistic regression model with 20,271 observations. *R*^2^ = .075. All models controlled for state-level alcohol policy score, age, gender identity, race, ethnicity, education, income, and parental status.

^b^ Odds ratios (ORs) represent the comparison between the listed groups and the No Delivery Allowed Group. Other significant differences between policy groups include the Expanded Delivery Group having higher odds of using alcohol delivery than the No Change in Delivery Group (OR = 1.22, 95% CI [1.09, 1.36]) and higher odds than the New Delivery Group (OR = 1.36, 95% CI [1.21, 1.52]).

### Average drinks per week and binge drinking days per month

Table 4 presents coefficients from regression models with average drinks/week and binge days/month regressed on time and DTC use. The model for average drinks/week included quadratic (*b* = 2.38, *p* = .001) and cubic effects of time (*b* = -0.72, *p* < .001) such that average drinks per week remained steady between pre- and early pandemic periods, gradually increased and peaked around the mid-pandemic, and then declined steeply (Supplemental Figure C). Using DTC delivery was associated with consuming 4.43 more drinks/week (*b* = 4.43, *p* < .001) compared with not using DTC delivery. There were no significant Time × DTC Use interactions.

**Table 4.**
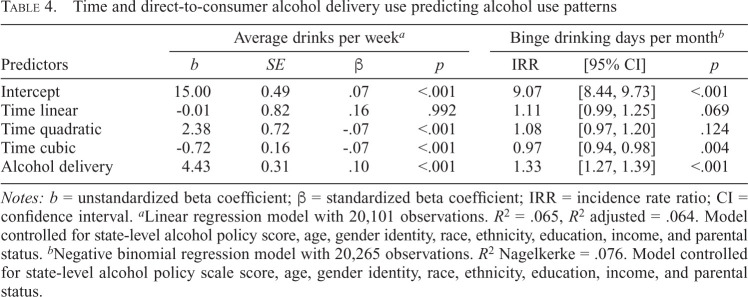
Time and direct-to-consumer alcohol delivery use predicting alcohol use patterns

Predictors	Average drinks per week*^a^*				Binge drinking days per month*^b^*		
	*b*	*SE*	β	*p*	IRR	[95% CI]	*p*
Intercept	15.00	0.49	.07	<.001	9.07	[8.44, 9.73]	<.001
Time linear	-0.01	0.82	.16	.992	1.11	[0.99, 1.25]	.069
Time quadratic	2.38	0.72	-.07	<.001	1.08	[0.97, 1.20]	.124
Time cubic	-0.72	0.16	-.07	<.001	0.97	[0.94, 0.98]	.004
Alcohol delivery	4.43	0.31	.10	<.001	1.33	[1.27, 1.39]	<.001

*Notes: b* = unstandardized beta coefficient; β = standardized beta coefficient; IRR = incidence rate ratio; CI = confidence interval.

^a^ Linear regression model with 20,101 observations. *R*^2^ = .065, *R*^2^ adjusted = .064. Model controlled for state-level alcohol policy score, age, gender identity, race, ethnicity, education, income, and parental status.

^b^ Negative binomial regression model with 20,265 observations. *R*^2^ Nagelkerke = .076. Model controlled for state-level alcohol policy scale score, age, gender identity, race, ethnicity, education, income, and parental status.

The negative binomial model for binge drinking days/month had a cubic effect of time (IRR = 0.97, 95% CI [0.94, 0.98]) with a similar pattern of change over time as average drinks/week (Supplemental Figure D). Using DTC alcohol delivery was associated with more binge drinking days/month (IRR = 1.33, 95% CI [1.27, 1.39]). There were no significant Time × DTC Use interactions.

### Negative drinking consequences

Table 5 presents coefficients from the zero-inflated negative binomial model with negative drinking consequences regressed on DTC use and average drinks/week. In the logit model predicting odds of experiencing at least one negative consequence, there was a significant positive interaction between DTC use and drinks/week (OR = 1.44, 95% CI [1.31, 1.58]). At levels of alcohol use less than an average of 1 drink per day (i.e., <6 drinks/week), DTC use was associated with a lower probability of experiencing at least one negative consequence compared with non-DTC use. For example, among people who consumed 2 drinks/week, DTC use was associated with a 5.3% probability of experiencing at least one negative consequence, compared with a 19.7% probability among people who did not use DTC. In contrast, among adults who consumed an average of 6 drinks/week or more, DTC use was associated with a higher probability of experiencing at least one negative consequence compared with non-DTC use. For example, among people who used DTC, drinking 10 drinks/week was associated with a 4.1% probability of experiencing at least one negative drinking consequence, compared with a 0.8% probability among people who did not use DTC. There was no significant interaction between DTC use and drinks/week. Using DTC delivery was associated with more negative drinking consequences (IRR = 1.28, *p* < .001), controlling for average drinks/week. Among people who used DTC, drinking 5, 10, or 15 drinks/week was associated with reporting 7.3%, 17.1%, and 27.9% higher incidence rates of negative consequences, respectively, compared with 1 drink/week. Supplemental Table D shows the sensitivity analysis without average drinks/week in the model.

**Table 5.**
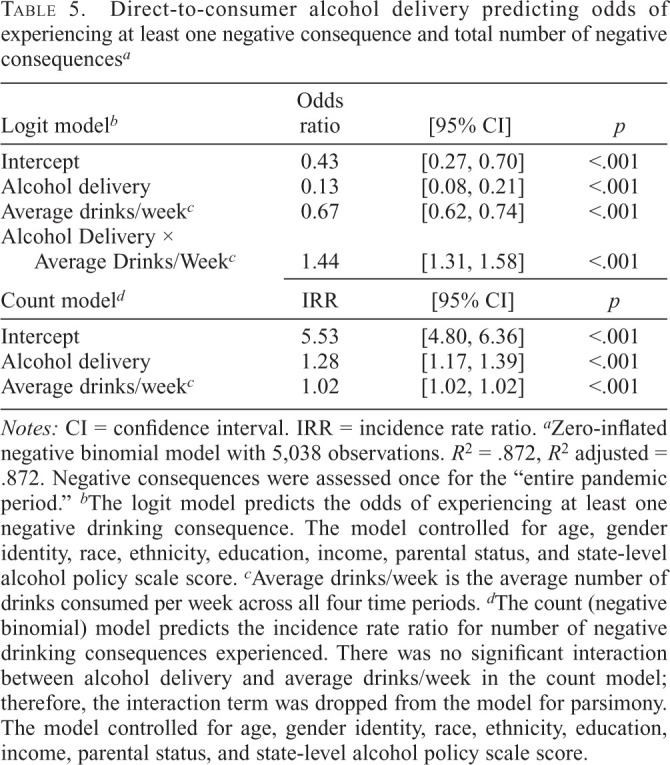
Direct-to-consumer alcohol delivery predicting odds of experiencing at least one negative consequence and total number of negative consequences*^a^*

Logit model*^b^*	Odds ratio	[95% CI]	*p*
Intercept	0.43	[0.27, 0.70]	<.001
Alcohol delivery	0.13	[0.08, 0.21]	<.001
Average drinks/week*^c^*	0.67	[0.62, 0.74]	<.001
Alcohol Delivery × Average Drinks/Week*^c^*	1.44	[1.31, 1.58]	<.001

Count model*^d^*	IRR	[95% CI]	*p*
Intercept	5.53	[4.80, 6.36]	<.001
Alcohol delivery	1.28	[1.17, 1.39]	<.001
Average drinks/week*^c^*	1.02	[1.02, 1.02]	<.001

*Notes:* CI = confidence interval. IRR = incidence rate ratio.

^a^ Zero-inflated negative binomial model with 5,038 observations. *R*^2^ = .872, *R*^2^ adjusted = .872. Negative consequences were assessed once for the “entire pandemic period.”

^b^ The logit model predicts the odds of experiencing at least one negative drinking consequence. The model controlled for age, gender identity, race, ethnicity, education, income, parental status, and state-level alcohol policy scale score.

^c^ Average drinks/week is the average number of drinks consumed per week across all four time periods.

^d^ The count (negative binomial) model predicts the incidence rate ratio for number of negative drinking consequences experienced. There was no significant interaction between alcohol delivery and average drinks/week in the count model; therefore, the interaction term was dropped from the model for parsimony. The model controlled for age, gender identity, race, ethnicity, education, income, parental status, and state-level alcohol policy scale score.

## Discussion

Our findings suggest that among the study sample, adults living in U.S. states that expanded DTC policies had higher odds of using alcohol delivery, and using DTC alcohol delivery was associated with greater average drinks per week, greater frequency of monthly binge drinking, and more negative drinking-related consequences. Our study goes beyond the previous studies that examined DTC alcohol delivery either before the pandemic (Fletcher et al., 2000; Mojica-Perez et al., 2019; Williams & Ribisl, 2012) or within the first year of the pandemic (Coomber et al., 2024; Grossman et al., 2022; Huckle et al., 2021; MacNabb et al., 2022; Trangenstein et al., 2023a, 2023b). The current study results provide information—among a large sample of U.S. adults—about DTC alcohol policies, typical DTC use, and alcohol-related patterns and negative consequences across several years.

Alcohol was not hard to access in the United States before the pandemic (Gray Phillip et al., 2018), with few states having restrictive alcohol policy environments (Blanchette et al., 2020). After the pandemic onset, the U.S. alcohol policy environment became less restrictive, with increasing access to alcohol via new or expanded DTC delivery policies (Colbert et al., 2021; Lemp et al., 2024; NIAAA, 2022; Trangenstein et al., 2023b). Similar to previous research (Grossman et al., 2020, 2022; Trangenstein et al., 2023b), we found that these policy changes corresponded with increased use of DTC alcohol delivery after the pandemic onset (March–May 2020), peaking mid-pandemic (June 2020–March 2021) and declining slightly thereafter. Individuals living in states with less restrictive policies had 22% to 111% higher odds of using DTC alcohol delivery. These policy changes expanded alcohol availability and accessibility despite evidence that greater availability generally corresponds with increases in drinking and alcohol-related harms (Babor et al., 2022; Community Preventive Services Task Force, 2023; WHO, 2019). Indeed, in an online convenience sample of 832 participants, 34.4% of adults cited increased alcohol availability as a reason underlying their increased drinking (Grossman et al., 2020).

We found that, compared with no DTC use, DTC use was associated with consuming greater than 4 more drinks/week and with a 33% increased rate of binge drinking days/month, corroborating previous research (Noel & Rosenthal, 2023; Trangenstein et al., 2023a, 2023b). These effects were larger than some previous studies with U.S. samples (Trangenstein et al., 2023a) and smaller than other U.S. and non-U.S. samples (Coomber et al., 2024; Grossman et al., 2022); differences are likely attributable to varying sample characteristics and the specific time frames examined. We found that the effect of DTC delivery on alcohol consumption did not change over time, suggesting that the effects of DTC use cannot be attributed solely to temporary increases in alcohol use during the COVID-19 pandemic (Barbosa et al., 2021; Grossman et al., 2020; Kerr et al., 2022; Leventhal et al., 2022; Patrick et al., 2022; Pelham et al., 2022; Pollard et al., 2020). Rather, our findings, in combination with previous research, indicate that using DTC alcohol delivery may be associated with overall increases in drinking. These findings can inform decision-making when future changes related to DTC home delivery policies arise by providing insights about some of the potential public health implications of these policies. To expand the surveillance of DTC policy effects on excessive alcohol use, jurisdictions could regularly collect and analyze data about alcohol purchasing and consumption behaviors (e.g., how people obtain their alcohol, type of alcohol purchased, and drinking patterns).

The public health impact of DTC delivery policies extends to alcohol-related consequences. This study found that, among all adults, using DTC delivery and consuming 5 or 10 drinks/week versus 1 drink/week increased the incidence rate of negative consequences by about 7% and 17%, respectively. Consistent with prior literature, these results indicate that using DTC delivery may contribute to individuals having more negative drinking-related consequences (Noel & Rosenthal, 2023; Trangenstein et al., 2023a, 2023b). Further research could evaluate whether the expanded use of DTC policies has been associated with changes in other measures of negative consequences, such as alcohol-related emergency department visits.

Many of the temporary DTC policy changes made in response to the pandemic—to initiate or expand DTC alcohol delivery—have become permanent, thereby increasing alcohol availability in many states (Colbert et al., 2021; Lemp et al., 2024). As of October 2021, a total of 46 U.S. states, comprising more than 95% of the U.S. population, allowed some kind of DTC alcohol sales with home delivery (NIAAA, 2022). Policy makers could strengthen alcohol delivery regulations and enforcement by implementing systems to monitor DTC alcohol delivery and limiting days and hours of alcohol deliveries, as is recommended for nondelivery alcohol sales (Community Preventive Services Task Force, 2023; WHO, 2019).

A systematic review indicated that regulations for online alcohol sales and delivery vary widely across jurisdictions, and many have lower levels of safeguards or ways to monitor compliance than brick-and-mortar alcohol establishments (Colbert et al., 2021). With fewer safeguards, DTC delivery businesses might not comply with other policies, such as evading alcohol taxes (Domenech et al., 2024; Lisa, 2024; Morton, 2021). In addition, DTC delivery could facilitate alcohol access and excessive drinking in underage individuals (Noel & Rosenthal, 2023; Williams & Ribisl, 2012). Noel and Rosenthal (2023) found that 12% of young adults in Rhode Island who ordered alcohol for home delivery or to-go never had their age verified, with 10% of purchases made by underage persons. Enhanced enforcement of laws prohibiting sales to minors, requiring age verification at time of purchase and delivery, and requiring responsible alcohol service training for workers about online DTC sales and delivery are potential strategies to address these concerns (Colbert et al., 2021; Community Preventive Services Task Force, 2023).

### Limitations

Although the sample composition was similar to the gender and racial composition of the general U.S. population, the sample included adults from 18 states, had fewer Hispanic adults, and had more adults who were more highly educated; therefore, results may not be generalizable (U.S. Census Bureau, 2022, 2023). The survey screening criteria were based on current state of residence, and it is possible that some participants lived in a different state before or during the pandemic. However, interstate migration rates were low across the study time frame (1.4%–2.2%), so it is unlikely that this affected results (Frost, 2023). Although the study examined DTC use and alcohol patterns during four periods, the cross-sectional design and the inability to randomly assign policy groups limit our ability to infer causality. Participants were asked about their typical drinking patterns across broad time frames, which precludes examining more discrete trends in DTC delivery and alcohol use patterns. The retrospective survey also introduced the risk for recall biases. However, the survey instrument and conditional logic were designed with these issues in mind, and quality control checks were used to counterbalance potential recall biases and overall data quality issues.

### Conclusions

Overall, among study respondents, this study suggests that living in a state that expanded their DTC policies corresponded with higher odds of using DTC alcohol delivery. The effects of DTC use were consistent across time, and using DTC alcohol delivery corresponded with greater alcohol use and higher levels of binge drinking across pre-, early, mid-, and late pandemic periods. DTC delivery use also increased the incidence of experiencing negative drinking-related consequences. These findings suggest that the expansion of permissive DTC alcohol delivery policies may inadvertently increase societal consequences related to alcohol use. Such data can inform states' future DTC alcohol policies.
